# Population Response to Habitat Fragmentation in a Stream-Dwelling Brook Trout Population

**DOI:** 10.1371/journal.pone.0001139

**Published:** 2007-11-07

**Authors:** Benjamin H. Letcher, Keith H. Nislow, Jason A. Coombs, Matthew J. O'Donnell, Todd L. Dubreuil

**Affiliations:** 1 S.O. Conte Anadromous Fish Research Center, United States Geological Survey, Leetown Science Center, Turners Falls, Massachusetts, United States of America; 2 Northern Research Station, United States Department of Agriculture (USDA) Forest Service, University of Massachusetts, Amherst, Massachusetts, United States of America; 3 Program in Organismic and Evolutionary Biology, University of Massachusetts, Amherst, Massachusetts, United States of America; Lund University, Sweden

## Abstract

Fragmentation can strongly influence population persistence and expression of life-history strategies in spatially-structured populations. In this study, we directly estimated size-specific dispersal, growth, and survival of stream-dwelling brook trout in a stream network with connected and naturally-isolated tributaries. We used multiple-generation, individual-based data to develop and parameterize a size-class and location-based population projection model, allowing us to test effects of fragmentation on population dynamics at local (i.e., subpopulation) and system-wide (i.e., metapopulation) scales, and to identify demographic rates which influence the persistence of isolated and fragmented populations. In the naturally-isolated tributary, persistence was associated with higher early juvenile survival (∼45% greater), shorter generation time (one-half) and strong selection against large body size compared to the open system, resulting in a stage-distribution skewed towards younger, smaller fish. Simulating barriers to upstream migration into two currently-connected tributary populations caused rapid (2–6 generations) local extinction. These local extinctions in turn increased the likelihood of system-wide extinction, as tributaries could no longer function as population sources. Extinction could be prevented in the open system if sufficient immigrants from downstream areas were available, but the influx of individuals necessary to counteract fragmentation effects was high (7–46% of the total population annually). In the absence of sufficient immigration, a demographic change (higher early survival characteristic of the isolated tributary) was also sufficient to rescue the population from fragmentation, suggesting that the observed differences in size distributions between the naturally-isolated and open system may reflect an evolutionary response to isolation. Combined with strong genetic divergence between the isolated tributary and open system, these results suggest that local adaptation can ‘rescue’ isolated populations, particularly in one-dimensional stream networks where both natural and anthropogenically-mediated isolation is common. However, whether rescue will occur before extinction depends critically on the race between adaptation and reduced survival in response to fragmentation.

## Introduction

Metapopulation theory predicts that the flow of individuals between subpopulations with different population vital rates is necessary for metapopulation persistence [1]. Under these conditions, habitat fragmentation and dispersal barriers should reduce abundance and population growth rates, increasing the risks of extinction. A considerable challenge to quantifying this extinction risk is integrating robust estimates of dispersal rates (necessary for understanding mechanisms) with detailed data on local demographic rates (necessary for making robust predictions of how dispersal affects local population dynamics).

However, not all populations function as metapopulations. Naturally isolated populations can persist in the absence of dispersal, and a large body of theory and empirical research has explored the conditions under which persistence is possible [2]–[4]. These isolated populations represent an extreme case along a continuum of subpopulation connectivity. Many species exhibit this entire range of conditions, from populations with high rates of subpopulation exchange to populations that are completely isolated. Therefore, a complete analysis of the demographic significance of connectivity needs to account for not just the effects of dispersal and local demography on persistence, but also an explanation of how isolated populations are able to persist.

Most metapopulation studies have focused on the ecological consequences of connectivity Given the reproductive isolation and potential for genetic drift in small isolated populations, a thorough understanding of the demographic consequences of connectivity also requires consideration of the potential evolutionary consequences [5], [6]. To do this, we need to determine first the spatial population genetic structure of the system, including the extent and time course of genetic differentiation among subpopulations. Also, we need to know how individual traits both influence and are influenced by dispersal probability in fragmented landscapes. For example, in species with high, size-dependent fecundity, changes in the vital rates (survival and growth) of large individuals may have disproportionately strong effects on population dynamics. If these large individuals are more likely to disperse, or if dispersal is a strong determinant of growth rate and size, dispersal restrictions will elicit a strong negative population response, potentially leading to local extinction. Further, if the reproductive success of large individuals depends on their ability to disperse, we would expect selection against large body size in isolated populations, and a consequent shift in size distribution.

Stream fishes in general, and stream salmonids in particular, possess attributes making them ideal model systems to study the importance of dispersal and fragmentation on population dynamics. These include constrained spatial distribution (within small stream channels) and dispersal (essentially one-dimensional dispersal along a stream network [7]), permitting high capture and recapture efficiencies. Further, this habitat configuration permits the effects of habitat fragmentation to be completely separated from the effects of habitat loss, which has been a major concern in habitat change studies [8]. Many stream systems are composed of “open” and “closed” populations (open populations are potentially connected through dispersal corridors, closed populations are naturally isolated by barrier falls). Finally, a portion of a freshwater salmonid population is also site-attached for much of its life cycle, allowing individuals to be followed more easily.

Fragmentation effects have important conservation and management implications, as extensive habitat fragmentation imposed by barriers to dispersal on streams (dams and road crossings) is thought to be a major threat to stream fish abundance and diversity [9], [10]. Several lines of evidence suggest that restricting movement along stream networks has negative effects on salmonid populations. Indirect evidence of the effects of fragmentation in streams comes from empirical studies which relate habitat patch size [3] or proximity to adjacent populations [4], [11] to probability of occurrence or abundance. In addition, Morita and Yokota [12] used a simple population model to define threshold population sizes necessary for persistence of white spotted charr (*Salvelinus leucomaensis*). For this same species direct evidence for increased extinction risk in small, isolated fragments was provided by Morita and Yamomoto [13] who found that probability of occurrence in stream fragments isolated by small dams increased significantly with fragment size. Similarly, for cutthroat trout (*Onchorhynchus clarki*) Harig and Fausch [14] found that the success of populations translocated to new habitats upstream of barriers was strongly dependent on habitat area upstream of the barrier. While these studies suggest that small salmonid populations are less likely to persist without supplementation by immigration, they leave important questions unanswered. First, there have been no studies on the effects of local demography and dispersal on network-wide persistence in stream fishes. Second, while isolated salmonid populations can persist [14], except for inbreeding depression in isolated populations [15], we do not know how isolation affects population vital rates, nor do we know how these effects contribute to population persistence.

In this study, we directly estimated size-specific dispersal, growth, and survival of stream-dwelling brook trout with a long-term, individual-based study. We used these data to develop and parameterize size-class and location-based population matrix projection models, allowing us to test effects of fragmentation on population dynamics at local and network-wide scales, and to identify demographic rates which influence the persistence of isolated and fragmented systems. Our linkage of intensive, long term data on population dynamics and movement rates with spatially explicit, stage-based projection models provides a general framework for understanding the demographic response to population fragmentation and isolation.

## Results

### (1) Reference matrix models

#### Model Goodness of Fit

Goodness of fit estimates indicated that the assumptions of multistate Capture-Mark-Recapture model we used to estimate transition probabilities from field data were not violated. An estimator of data overdispersion, c-hat (values<2 indicate no overdispersion [16]) indicated no assumption violations for either the Open system (0.94) or the isolated tributary (1.2).

#### Open system

A stage 0 survival (the only model parameter not directly estimated from the data) of 0.0336 generated a λ of 1 (Table 1). Parametric bootstrap resampling of the reference matrix yielded a 95% confidence interval range for λ of 0.990 to 1.012 with an average of 1.0008. Stable stage distributions indicated that size class zero would contain the most fish (75%), followed by size class 4 (13%) and size classes 1–3 (each about 4%) (Figure S1). Generation time equaled 1.91 years. Monthly survival averaged over locations decreased slightly from size class one to four (0.93, 0.91, 0.91, 0.90). The largest elasticity (greatest influence on variation in λ) was for survivals of size class four fish that remained in the same location (Table S1).

**Table 1:** Reference matrix describing monthly size- and location-based survivals and fecundities (F in rows 1–3) for the West brook (WB) and OpenSmall (OS) and OpenLarge (OL) tributaries.

		WB0	OS0	OL0	WB1	WB2	WB3	WB4	OS1	OS2	OS3	OS4	OL1	OL2	OL3	OL4
		1	2	3	4	5	6	7	8	9	10	11	12	13	14	15
F WB	1	0	0	0	0.678	1.356	2.138	4.428	0	0	0	0	0	0	0	0
F OS	2	0	0	0	0	0	0	0	0.669	1.345	2.123	4.492	0	0	0	0
F OL	3	0	0	0	0	0	0	0	0	0	0	0	0.757	1.541	2.516	4.442
WB1	4	0.03356	**0**	**0**	0.397	**0**	**0**	**0**	*0*	**0**	**0**	**0**	0.018	**0**	**0**	**0**
WB2	5	**0**	**0**	**0**	0.360	0.412	**0**	**0**	0	0.011	**0**	**0**	0.013	0.009	**0**	**0**
WB3	6	**0**	**0**	**0**	0.132	0.381	0.479	**0**	0	0.046	0.032	**0**	0	0.016	0.016	**0**
WB4	7	**0**	**0**	**0**	0.004	0.069	0.376	0.839	0	0	0.068	0.223	0	0	0	0.025
OS1	8	**0**	0.03356	**0**	0	**0**	**0**	**0**	0.393	**0**	**0**	**0**	0	**0**	**0**	**0**
OS2	9	**0**	**0**	**0**	0.001	0.005	**0**	**0**	0.431	0.409	**0**	**0**	0	0	**0**	**0**
OS3	10	**0**	**0**	**0**	0.001	0.003	0.008	**0**	0.146	0.433	0.455	**0**	0	0	0	**0**
OS4	11	**0**	**0**	**0**	0	0.006	0.008	0.026	0.013	0.044	0.381	0.663	0	0	0	0.032
OL1	12	**0**	**0**	0.03356	0.006	**0**	**0**	**0**	0	**0**	**0**	**0**	0.484	**0**	**0**	**0**
OL2	13	**0**	**0**	**0**	0.002	0.009	**0**	**0**	0	0	**0**	**0**	0.344	0.542	**0**	**0**
OL3	14	**0**	**0**	**0**	0.003	0.005	0.007	**0**	0	0	0	**0**	0.037	0.345	0.668	**0**
OL4	15	**0**	**0**	**0**	0	0.001	0	0.022	0	0	0	0.027	0	0	0.225	0.835

The numbers following location designations refer to size categories (see text for definition). Bold entries represent impossible transitions that were fixed to 0 and underlined 0's represent transitions estimated to be 0.

Reference matrix projections predicted significant variation among locations in key demographic parameters. Monthly survival averaged over size classes was lowest for fish that began a sampling interval in the WB (0.89), was highest for OS (0.94), and was intermediate for OL (0.90, Table 1). Concordant with total habitat area, the WB was predicted to contain the largest percentage of the total population (60%), followed by OL (28%) and OS (12%) tributaries. Among locations, elasticities were generally greatest for WB, intermediate for the OL, and smallest for OS (Table S1).

The direction and magnitude of movement varied with location and fish body size. Fish were much more likely to leave a tributary than to enter a tributary from the WB. This was especially true for OS where the ratio of the probability of leaving summed over size classes to the summed probability of staying was 6.2 (0.39/0.06), compared to 2.2 (0.22/0.10) for OL. On average, about one-half of the probability of movement in either direction could be attributed to fish from the largest size class, except for OL where the probability of leaving was more evenly spread across size classes (Table 1). Movement between tributaries was rare, but did occur for fish from the largest size class (Table 1).

#### Isolated tributary-Open system comparison

**Figure 1:**
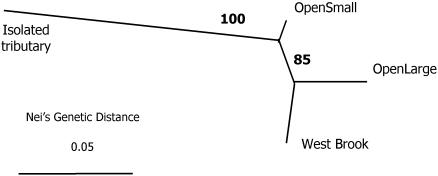
Population genetic structure among the Open system and the Isolated tributary. Numbers represent the percentage of bootstrap runs supporting the tree structure.

Population genetic results indicated that the Isolated tributary was genetically distinct from the Open system (Figure 1). Comparison with hatchery fish indicated no measurable introgression into either wild population (bootstrap value = 100%). The estimated time since divergence of the Isolated tributary from the Open system was 455 (95% C.I. 348–609) generations or approximately 910 (698–1218) years (based on a generation time of two years). Effective population sizes (N_e_) were Isolated = 91.9 (69.6–125.5), OS = 29.3 (25.2–33.3), and OL = 113.1 (93.1–140.7). We were unable to estimate N_e_ for WB due to incomplete sampling.

**Figure 2:**
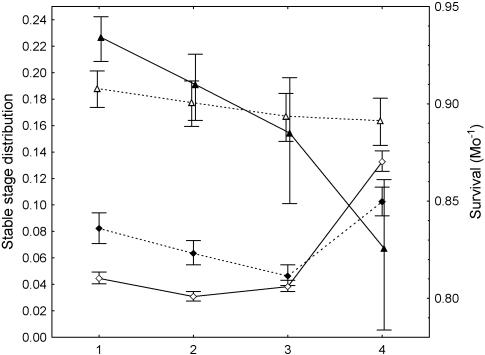
Stable stage distributions (diamonds; ±95% CI) and survival (triangles; ±95% CI) for the Isolated tributary (closed symbols) and for the summed size stages across locations in the Open system (open symbols). Stage 0 data are omitted for clarity.

In general, demographic variables in the isolated tributary indicate a strong shift towards the importance of smaller fish compared to the Open system. A stage 0 survival of 0.0488 generated a λ of 1 (Table 2) for the Isolated tributary, which was 45% higher in the Isolated tributary compared to the Open system. This difference appeared insensitive to the assumption that λ = 1 (Figure S2). Parametric bootstrap resampling of the reference matrix yielded a 95% confidence interval range for λ of 0.978 to 1.020 with an average of 0.9996. Generation time in the Isolated tributary (0.83 years) was about one-half of that in the Open system. Stable stage distribution differences reflected the shift to more stage 1 and stage 2 fish and fewer stage 4 fish in the Isolated tributary compared to the Open system (Figure 2). Finally, survival was strongly size-dependent in the Isolated tributary, with considerably higher survival for smaller fish, but did not vary across size in the Open system (Figure 2).

**Table 2:** Reference matrix describing monthly size-based survivals (1–4, see text for definition) and fecundities (F in row 1) for the Isolated tributary.

		1	2	3	4
F	0	0.815	1.551	2.185	4.273
1	0.04875	0.583	**0**	**0**	**0**
2	**0**	0.342	0.558	**0**	**0**
3	**0**	0.010	0.341	0.516	**0**
4	**0**	0	0.011	0.369	0.826

Bold entries represent impossible transitions that were fixed to 0 and the underlined 0 represents a transition estimated to be 0.

**Figure 3:**
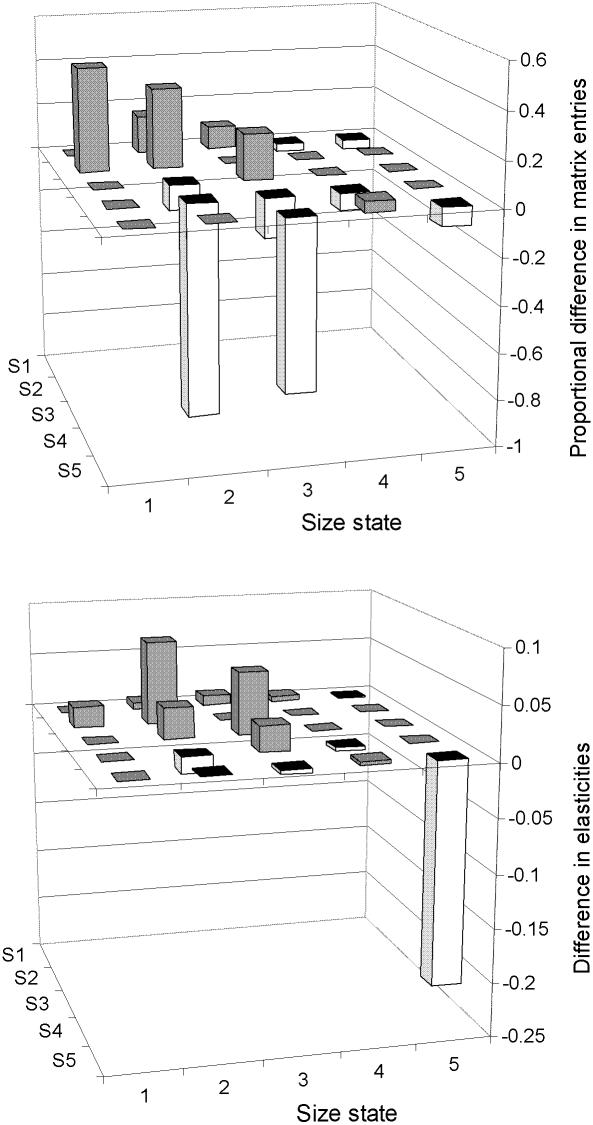
Proportional difference in matrix entries (above) and difference in elasticities (below) between the Isolated tributary and the Open system (values collapsed over locations for the Open system). Positive values (closed bars) represent higher matrix entries or elasticities for the Isolated tributary.

Direct comparison of matrix entries clearly reflected the greater importance of smaller fish in the isolated tributary compared to the Open system; survivals for non-growing fish were 20–35% higher for stage 1 and 2 and 7–8% lower for stages 3 and 4, and transitions for surviving and growing into the next stage were 10 to 91% lower (Figure 3). Absolute differences in elasticities (see Table S2 for Isolated tributary elasticities) also reflected the importance of smaller size stages in the Isolated tributary compared to the Open system (Figure 3). Elasticities for surviving and remaining in stages 1 and 2 were 0.06 and 0.08 greater in the Isolated tributary while the elasticity for surviving in stage 4 was much higher (0.21) in the Open system.

### (2) Effects of simulated fragmentation

#### Tributary extinction times

**Figure 4:**
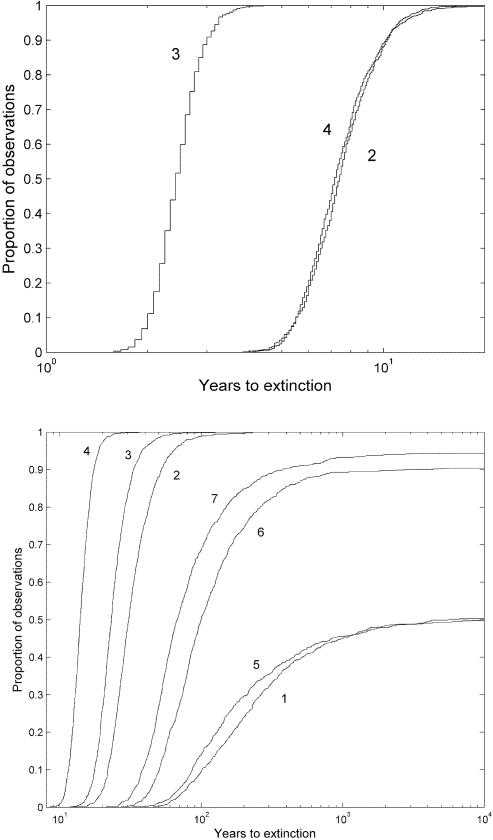
Empirical cumulative distributions of years to tributary extinction (above) for the three tributary scenarios and system extinction (below) for the seven scenarios. Scenario identifiers are in Table 3. Distributions were based on 1000 parametric bootstrap samples for each scenario.

Blocking entry of fish from the mainstem resulted in rapid (∼2–6 generations) predicted extinction times for both of the currently-open tributaries (Figure 4). This effect was most extreme for blocking access to OS only, where extinction was predicted within 2.9 years (90% confidence) or 3.2 years (95% confidence). In simulations where access to either OL or to both OL and OS was blocked, extinction was predicted within 10.1 years (90% confidence) or 11.2 years (95% confidence)(Figure 4).

**Table 3:** Average and confidence intervals for λ, the percentage of runs with λ<1, and the number of years to extinction for two probability levels (see Figure 4) based on 1000 parametric bootstrap samples for the seven scenarios (reference matrix and the six fragmentation scenarios) for the Open system.

	Scenario	Average λ [95% C.I.]		Percent of runs with λ<1	Number of years to system extinction		Number of immigrants per year for λ = 1	Proportion of initial population size immigrating per year for λ = 1
					90%	95%		
1	Reference	1.0008	[0.9903; 1.0115]	50.3	-	-	-	-
2	Remove OL	0.9815	[0.9664; 0.9956]	100	52.2	63.2	295.9	0.20
3	Remove OS	0.9774	[0.9635; 0.9902]	100	33.0	38.0	408.8	0.27
4	Remove Both	0.9612	[0.9446; 0.9770]	100	17.2	18.9	688.1	0.46
5	Redistribute OL	1.0001	[0.9861; 1.0131]	49.8	-	-	-	-
6	Redistribute OS	0.9944	[0.9824; 1.0067]	90.4	2349.3	-	100.9	0.07
7	Redistribute Both	0.9918	[0.9773; 1.0072]	94.4	377.7	-	135.4	0.09

Also shown is the number and proportion of immigrants required to ‘rescue’ the system from extinction (λ = 1).

#### Open system extinction

Blocking access to the tributaries decreased overall population growth rates and increased the likelihood of network-wide extinction. The likelihood and timing of system extinction depended on whether fish were removed or redistributed and on which tributary was blocked. When fish that were blocked entry to a tributary were removed from the population, system extinction occurred in 100% of the bootstrap runs, reflecting the low average λ values and lack of 95% confidence interval overlap with a λ of 1 (Table 3). In removal scenarios, system extinction was predicted to occur most rapidly (within 17 years at 90% confidence) when access to both tributaries was blocked. When only one of the tributaries was blocked, blocking OS resulted in more rapid whole-system extinction (within 33 years with 90% confidence) than blocking OL (within 52 years at 90% confidence). (Figure 4, Table 3).

System extinction was less likely for the redistribution scenarios than for the removal scenarios. Redistributing fish when the OL tributary was blocked had very little effect on the likelihood or timing of extinction compared to the reference case (Figure 4, Table 3). In contrast, blocking access to both tributaries resulted in system extinction in 94% of the runs, within 378 years at 90% confidence. Blocking access to the OS tributary generated intermediate values; 90% of the runs resulted in system extinction which was predicted to occur within 2349 years with 90% confidence.

#### Rescue by immigration

The minimum number of immigrants required to ‘rescue’ the Open system and prevent extinction (return λ to 1 following fragmentation) reflected probabilities of extinction, with more immigrants required when system extinction was more likely to occur quickly (Table 3). For the scenario with the shortest time to system extinction (Remove Both), 688 fish per year, or 46% of the population, would be required to eliminate chances of system extinction. In contrast, the scenario with the longest time to system extinction (Redistribute OS) needed 101 fish per year, or 7% of the population, to prevent extinction due to fragmentation.

#### Rescue by demography

Incorporating the stage 0 survival from the Isolated tributary into the Open system increased λ and reduced extinction risk. On average, λ increased 3.2% following incorporation of the Isolated tributary stage 0 survival. This change in early survival rate ‘rescued’ the Open system except in the most extreme case of blocking access to both tributaries and removing the blocked fish (Table S3). In this case, 90% of the runs resulted in system extinction within 337 years (90% confidence level, data not shown), compared to extinction within 17 years with the lower Open-system survival rates (see above). For the remaining fragmentation scenarios, system extinction was extremely unlikely with the higher stage 0 survival rate (Table S3).

## Discussion

We found that fragmentation, independent of habitat loss [8], increased extinction risk in a stream network. Although the importance of fragmentation in stream networks has been suggested by other lines of evidence [13], [17] it has not been previously demonstrated. These results confirm the prediction that movement may be particularly important to population persistence in branching networks habitat [7]. These results also confirm a key prediction of metapopulation theory in a stream system, indicating that species persistence at the network scale depends on movement of individuals among sites. However, we also found that the naturally-isolated subpopulation persisted despite a complete lack of immigration. Persistence in this case was associated with two key differences–lack of emigration and a dramatic shift in demographic rates. These differences suggest that local adaptation at small spatial scales may play an important role in maintaining small isolated populations in stream networks.

Isolation of previously-connected terminal nodes (tributaries) in stream networks can increase the probability of tributary extinction. Previous studies suggest that tributary size is a key determinant of extinction probability following isolation [13], [14]. In these studies, tributary size is assumed to correlate directly with population size, with larger populations in larger tributaries more resilient to stochastic population fluctuations and environmental variability. In our study, extinction probability (years to predicted extinction) also correlated with tributary and population size. Consistent with these previous results, the OS tributary, which had the smallest N_e_ and the least amount of habitat, had the shortest time to extinction following simulated fragmentation (one-fifth the time as the larger open tributary). However, this tributary had the highest rates of emigration to the mainstem, which may exacerbate vulnerability to local extinction.

In spite of the negative effects of population fragmentation, isolated populations do persist under some circumstances. In our study, brook trout in one tributary have been isolated from the mainstem for>400 generations. In addition to being genetically distinct, this population differs demographically from the open tributary/mainstem population. At stable size distributions, brook trout in this isolated population have significantly higher early survival and reproduce at smaller size stages than the open population, resulting in a size distribution skewed toward smaller individuals. Differences in size distributions and mortality schedules may represent a phenotypic response to different environmental conditions [18]. Alternatively, these differences may represent local adaptation, if there is heritable variation for, and strong selection on, traits such as body size. Supporting a genetic basis, we did observe a large difference in viability selection on size between the isolated and tributary mainstem populations. In stream salmonids, growth rate generally has high heritability [19], [20]. This combination suggests that differences could result in local adaptation, and rapid evolution in demographic traits has been demonstrated in fish populations [21]–[24]. More generally, changes in the size distributions of isolated populations have been well documented in the ecological literature [25] although the directions of these changes (increases vs. decreases) may differ among species and systems.

Our results further suggest that the demographic characteristics of the isolated population contribute to persistence. When the early survival rates of the isolated population were applied to the tributary/mainstem population, it was rescued from extinction in most fragmentation scenarios. Life history theory predicts that higher early survival and earlier maturation increases resilience to stochastic extinction [26]. If these demographic characteristics have a genetic basis, local adaptation may play an important role in the persistence of isolated populations. Essentially the question becomes, will populations evolve demographic characteristics that will enable persistence before the population goes extinct [27]? This question is important for conservation and management. Fragment size is a critical component, particularly as the number of barriers in a stream network increases. The probability of evolving demographic characteristics in time to stave off extinction will decrease with fragment size, as these fragments have less time before hitting zero, and potentially less genetic variation for selection to work with. Further, these results suggest that even when extinction does not occur, fragmentation may result in the loss of important population characteristics, for example large body size and movement strategies. Further progress in the integration of demography and evolution will allow more precise determination of extinction dynamics in these systems, as well as contributing to our general understanding of the links between the evolution and ecology of spatially-structured populations.

In addition to increasing the extinction probabilities in tributaries, fragmentation in some scenarios increased extinction probability of the whole population (mainstem plus connected tributaries). Previous studies have documented that individual fish readily move from mainstem to tributaries, and that small tributaries, by virtue of the structure of bifurcating stream networks, may provide habitat for a large proportion of the individuals in a population [7]. However, no previous studies have quantified the effect of tributary isolation on combined tributary/mainstem dynamics. In our simulations, blocking access to tributaries increased their likelihood of extinction, which in turn increased the likelihood of extinction of the whole system. Given the high proportion of stage-0 fish in the tributaries, it appears that open tributaries in this system act as reproductive sources. Fish enter the tributaries to spawn, use them as nurseries during stage-0, with some proportion leaving as they grow. However, all tributaries are not equal in their importance to system-wide persistence. Blocking access to the tributary with the smallest effective population size, but the highest rates of emigration to the mainstem had the largest impact on whole-system extinction. This small population is an importance source (22% of the large fish produced here leave), but is highly vulnerable to isolation resulting in the rapid loss of this source under fragmentation scenarios. In contrast, isolating the larger open tributary, with nearly 4× the effective population size, but much lower emigration rates, had a smaller impact on system-wide extinction, largely because this subpopulation is less dependent on immigration and can persist longer when isolated. These results suggest that while subpopulation/habitat size may be a strong determinant of local persistence [13], [14], understanding the response of stream networks to fragmentation requires accounting for both habitat size and movement rates [28].

In our study, the effects of tributary isolation on the whole population depended on the fate of those individuals that were prevented from entering tributaries from the mainstem. Because the magnitude of the costs associated with different fates is difficult to determine, we bracketed the potential costs between two extremes: complete cost (removal) and no cost (redistribution). In our study, imposing complete costs had major negative effects on whole-system persistence. With no costs, negative effects on persistence were smaller, but under some scenarios, fragmentation still increased extinction probability of the whole system. In reality, the actual costs to individuals that are blocked from tributaries must lie between these extremes, and the response to fragmentation will depend on the magnitude of density-dependent growth and survival and increased competition for appropriate habitats. There is a large body of evidence that growth and survival are strongly density- and habitat-dependent in stream salmonids [e.g. 29], and therefore the inability of individuals to disperse from high-density conditions and search effectively for appropriate habitat should have some costs. Incorporation of these dynamics into projection models, along with continued advances in our understanding of density-dependence and habitat selection, will be instrumental in future analyses.

A key component of metapopulation theory is the rescue of fragmented populations by immigration from outside the system [1]. In general, for species inhabiting branching networks such as streams, there are generally ‘downstream’ or ‘upstream’ limits to species distributions, caused by longitudinal gradients in habitat conditions. For example, brook trout are limited in their downstream distribution by temperature, substrate and dissolved oxygen requirements. These requirements will limit the ability of rescue from downstream, dependent on the position of the study system in the stream network. Further, these observations suggest a predictable upstream increase in the vulnerability of fragmented populations in stream networks. In our study, incorporating immigration from downstream of the study area was observed to rescue the tributary/mainstem population from extinction resulting from fragmentation under most scenarios. A major strength of our approach is the ability to quantify the required immigration rates, which can then be used to determine whether this rescue effect is likely. In our system required immigration rates were generally much higher than observed immigration (<15% of the total population), limiting the ability of this mechanism to reduce extinction probability. These results further underscore the utility of our approach using frequent sampling of identifiable individuals, robust estimates of individual movements, and an analytical framework for estimating size- and location-dependent survival.

## MATERIALS AND METHODS

### Study Species and Site

Brook trout (*Salvelinus fontinalis*) are native to the eastern United States and are present in most small coldwater stream habitats not heavily impacted by acid rain or acid mine drainage [30]. Brook trout are iteroparous, have a strong, positive fish size-fecundity relationship, and males mate with multiple females in the autumn. Females deposit eggs in gravelly stream-bottom nests that hold the developing embryos over winter from which fry emerge in late winter/early spring. Maximum age in our study area is four years (Letcher et al. unpublished data).

**Figure 5:**
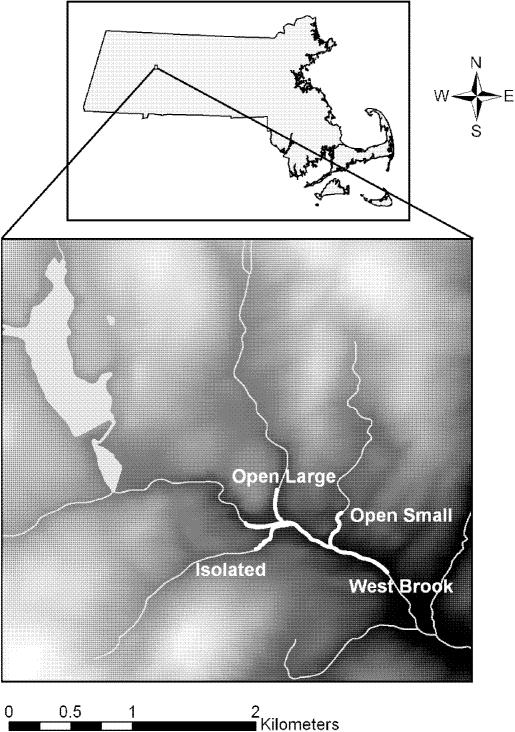
Map of the study area watershed in western Massachusetts, USA. Study area indicated by bold white lines.

Our study area (42°25′N, 72°39′W) consisted of a 1-km-long mainstem (West Brook, abbreviated WB) with two accessible second-order tributaries (Jimmy Nolan Brook [hereafter termed OpenLarge and abbreviated OL] and Mitchell Brook [OpenSmall, OS]) which we collectively refer to as the Open system. In addition, an inaccessible second-order tributary (Ground Brook [Isolated]; southern tributary in Figure 5) represented our Isolated tributary.

Tributary study area lengths were 300±5 m with waterfalls blocking upstream fish passage at the upstream end of each study reach. A 2.2-m tall waterfall blocked access to the Isolated tributary from the WB. Over four years of possible detection of emigration from the Isolated tributary (PIT tag antenna placed at the confluence), less than 0.1% of the Isolated population has been observed moving from the Isolated tributary to the WB and none have entered the Isolated tributary from the WB. Average stream width was widest for the WB (4.5 m), intermediate for OL (3 m) and narrowest for OS (2 m) and Isolated (2 m). The stream habitat consisted mainly of cobble and riffles with several pools (fewest in OS) and the riparian zone was forested with a well developed canopy (mixed hardwoods). In addition to brook trout, the WB, OL and OS contained naturally reproducing non-native populations of brown trout (*Salmo trutta*), and Atlantic salmon (*Salmo salar*) were stocked as fry (∼26-mm) each spring into the WB (50·100 m^−2^). Trout from hatcheries were not stocked into the study area during the course of the study. A pair of stationary tag-detecting antennas was placed at the bottom of the study site to detect permanent emigrants (91% average detection efficiency [31]).

### Sampling

In each year of the study (2001–2006), we sampled fish on three or four occasions (spring, summer, autumn, winter) throughout the study area. Fish were captured using standard electrofishing techniques (400 V DC, unpulsed). During sampling, we made two passes through 20-m long stream sections that were isolated using temporary block nets. Captured fish were measured for length (fork length) and untagged fish>60 mm [32] were tagged with 12 mm passive integrated transponder tags (PIT tags, Digital Angel, St. Paul, MN, USA) following anesthesia with clove oil (30 mg·L^−1^). All sampling was conducted in accordance with the USGS Conte Anadromous Fish Research Center's animal care and use protocols.

### Analyses

We report data from three brook trout cohorts (2001–2003, age-0+ in autumn of year) over the course of 16 sampling occasions. For the three cohorts, 834 (2001), 719 (2002), and 971 (2003) fish were available for analysis. We conducted two sets of analyses based on the body size- and location-based population projection matrix [see 33] for the Open system (including the WB, OL, and OS populations) and on the body size only matrix for the Isolated tributary. (1) We used the model to examine basic demographic variables of the systems and to compare demographic variables between the Open system and Isolated tributary. (2) We examined the effects of simulated fragmentation by altering the basic matrices for the Open system. We estimated Open system (WB, OL and OS) and tributary (OL and OS independently) extinction times under simulated isolation of the tributaries. We also estimated numbers of immigrants required to ‘rescue’ the system from extinction and explored whether the Open system could be ‘demographically rescued’ from simulated fragmentation. In addition, we estimated genetic distance and divergence times between the Isolated tributary and the Open system to provide an indication of the degree of genetic population structure.

#### (1) Reference matrix models

**Figure 6:**
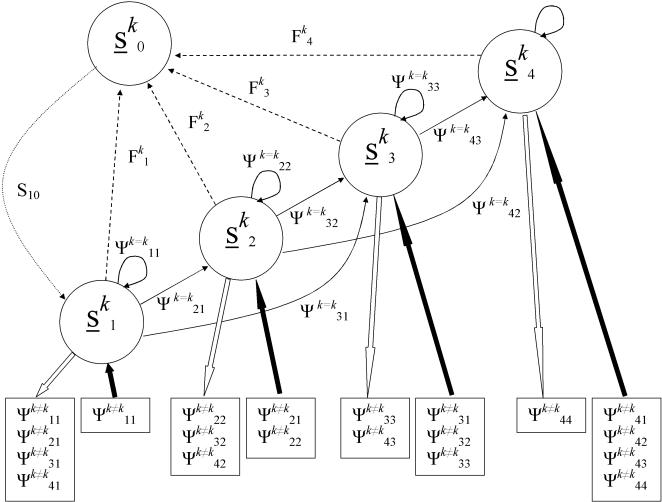
Graphical representation of the life history and spatial transition model. For simplicity, the full life history model with each size state (s) is shown for a single location (*k*) only. Transitions from size states *j* to *i* are represented by Ψ*^k = k^_ij_* within a location and fecundities for each size state are represented by F*^k^_s_*. The only parameter not estimated from field data was survival from size state 0 to size state 1 (S_10_). Transitions between locations Ψ*^k≠k^_ij_* are listed in boxes for individuals leaving (open arrows) or entering river *k* (closed arrows).

The reference matrix models contained three classes of parameters, that each required different parameter estimation approaches. First, we used multi-state capture-mark-recapture models [34], [35] to generate parameter estimates [36] for the transitions between combinations of the location (three for the Open system, one for the Isolated tributary) and size (four states) states (see details below and Figure 6). Second, size-based fecundity estimates were obtained from field samples. We estimated a fish size (x, mm), fecundity (y, number of eggs) relationship (y = 0.00187·x^2.190^, r^2^ = 0.64, N = 40) that we used to generate fecundity estimates for the midpoint of each size state. Field samples indicated an non-significant interaction between tributary and size (ANCOVA, P = 0.42), consequently the same relationship was used for all locations. Third, the only parameters for which we do not have direct estimates are location-specific survival from egg to first tagging (age-0 autumn). For the Open system matrix, we estimated a common survival for this early survival stage (coded as size state ‘0’) across locations that provided a population growth rate (λ) of one. For the Isolated tributary, we estimated an independent early survival stage survival that generated λ = 1 for the isolated tributary matrix. To assess the sensitivity of our early survival estimates to the assumption that λ = 1, we also estimated early survival for λ values ranging from 0.9 to 1.1.

### Parameter Estimation

We constructed a body size- and location-based matrix projection model using our field data to serve as the foundation for the Open system matrix model. For this system, the locations were the three stream network segments (West Brook and the two Open tributaries, *k* = [1], [2], [3] in Figure 6). For the Isolated tributary, we generated separate parameter estimates across body sizes for the single location (*k* = [1] in Figure 6). For both systems, fish sizes were divided into four approximately equally represented fish size bins (mm, fork length; 60–95, 95–115, 115–135, >135). These bins also roughly corresponded with age in autumn, although there is considerable overlap in age categories for fish larger than 115 mm. The combination of three locations and four sizes yielded 12 possible states for the Open system and one location and four size states yielded four possible states for the Isolated tributary. We estimated transition probabilities (Ψ_ij_) (from state j to state i) using a multistate capture-mark-recapture model [34], [35]. Input to the model was individual capture histories coded for states of 1–12 depending on location and size at the capture occasion for the Open system and 1–4 depending on size alone for the Isolated tributary. Individuals not captured on an occasion were assigned a state of ‘0’ in the input file encounter history and permanent emigrants from the WB or the Isolated tributary were assigned a frequency code of ‘−1’.

We used Program M-Surge [37] to obtain parameter estimates. The number of parameters to estimate for multistate models can be very large, depending on the number of states and the complexity of the model. For the Open system, the most general model we could use to generate the transition parameters for the matrix contained at most 145 parameters (12·12+1 parameter to account for probability of capture). Fortunately, many (48) of the parameters could be fixed to zero because they described impossible transitions (fish do not shrink in length, see unlisted Ψ*^k≠k^_ij_* in Figure 6 and shaded entries in Table 1). For simplicity, we did not estimate parameter variation in time or among cohorts (both vastly increase the number of parameters to estimate). We used program U-Care [38] to estimate goodness of fit for our data to the multistate model. We report the summed chi-square values and P-values for the multistate tests available in U-Care.

The real parameter estimates provided by multistate models must be converted before they can be incorporated into a matrix model. All Ψ_ij_ must be multiplied by S_j_, the probability of survival given that an individual began the sampling occasion in state j. thus, transition entries in the matricies represent the probability of transitioning given survival over the sampling interval. By default, M-Surge constrains 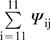, i≠j, to a value of less than or equal to one. The time unit of S was monthly, corresponding with the time scale of sampling.

We built two-sex matrix projection models because we cannot identify sex of all fish. We assumed a 50∶50 sex ratio (chi-square P = 0.23, based on 40 known sex individuals). Accordingly, we multiplied the fecundity values obtained from the fish size-fecundity relationship by 0.5. To scale the fecundity entries to the monthly survival estimates, we also divided fecundities by 12. Although this is clearly unrealistic, it does not affect results of this model because we are not examining within-year effects. Finally, we multiplied the fecundities by the square root of the summed survival estimates for fish of each size class to represent our assumption that fish survived on average one-half of a sampling interval before spawning.

For both systems, we present standard demographic variables for the reference matrices including stable stage distribution, elasticities and generation times [33]. Parametric bootstrap was used to generate distributions around λ (details in Supplemental Text S1).

#### 

##### Isolated tributary-Open system comparison

To provide genotypes to estimate genetic distance and divergence time between the Isolated tributary and the Open system, we genotyped a total of 1712 individuals from the 2001–2003 cohorts at 12 microsatellite loci ([39], Tim King, USGS Leetown, VA unpublished data). In addition, we genotyped 20 hatchery fish to assess the potential for historical introgression of stocked fish (hatchery fish were stocked into the system historically). Genetic distances for the five populations were calculated using Nei's measure [40] in program PHYLIP [41]. A neighbor-joining tree using the method of Saitou and Nei [42] was constructed, along with 1000 bootstrap replicates to assess tree congruence.

To acquire an estimate for divergence time (T) between the isolated tributary and open system populations, we used program BATWING [43]. We ran the scaled model and set the prior distribution for θ (4N_e_μ) to be uniform. Runs consisted of a burn-in period of 20,000 steps followed by a run of 200,000 steps. A total of eight runs was conducted: four using twenty individuals from the isolated tributary and twenty individuals divided equally among the open system populations, and four using sample sizes of forty individuals. Samples were randomly chosen from the populations. The final estimate of T and its 95% confidence interval were derived from the estimates produced by the eight runs. To convert T into units of generations and years we multiplied by the effective population size (N_e_) summed over all populations, and a generation time of two years. Population-specific N_e_ were estimated from genetic data using program MLNe [44], [45].

To compare demographic estimates of the Isolated tributary with those from the Open system, we compared the matrix entries and demographic variables of the Isolated tributary matrix to a size-only matrix for the Open system (estimates collapsed over location). First, we estimated means and confidence intervals for λ and stable stage distributions for the Isolated tributary using the parametric bootstrap approach outlined in Supplemental Text S1. Next, we collapsed values for the Open system in several ways. For elasticities, we simply summed values across locations for each size transition or size state (fecundities). For the size-based estimates which were generated with the parametric bootstrap, stable stage distributions were summed across locations for each size state for each bootstrap realization. Then, means and 95% confidence intervals were calculated for each size state. For matrix entries themselves, we first summed across possible transitions within each size and location combination (yielding 48 values) and then averaged across locations (yielding 16 values) to provide size-based transitions. We also averaged over location-specific fecundities to provide size-based fecundities.

We compared Isolated and Open system matrix entries by examining proportional changes in each matrix entry (Isolated/Open −1). Differences in elasticity were represented as the difference between Isolated tributary values and Open system values for each matrix entry. We also compared means and 95% confidence intervals for stable size distributions for each size state.

#### (2) Effects of simulated fragmentation

### Simulating fragmentation

We simulated fragmentation in the Open system by altering the basic matrix to block entry of fish that would have otherwise entered into either or both tributaries (fragmentation in stream systems often blocks upstream passage, but not downstream passage). Transitions for departure from tributaries were left unaltered. Entry was blocked by setting all transitions into the tributary to 0 (i.e., for OL the intersection of rows 12–15 and columns 4–11 in Table 1; for OS rows 8–11 and columns 4–7 and 12–15).

The fate of fish that would have entered tributaries is unknown, so we simulated two extreme forms of density dependence for these fish; either redistributing the fish among the other transitions (no density dependence) or removing the fish (extreme form of density dependence). When fish were redistributed, we used
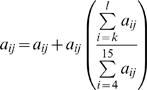
(2)where *a_ij_* was the matrix entry for row *i* and column *j*, and *k* and *l* indicated rows for transitions into OL (*k* = 12 and *l* = 15) or OS (*k* = 8 and *l* = 11). Stage 0 matrix entries (first three columns in Table 1) were not altered. When fish were removed, *a_ij_* that were not set to 0 remained unaltered. We simulated a total of six fragmentation scenarios- the combination of the two density dependence scenarios (Remove and Redistribute) and the three blocked entry scenarios (OL blocked, OS blocked, both blocked).

### Extinction time

Extinction was defined as the presence of<2 individuals. For the whole system analyses, extinction occurred when<2 individuals remained in the entire Open system. For the tributary dynamics analyses, extinction occurred when<2 individuals remained in a single tributary (OS or OL). We defined extinction as fewer than two individuals to be as conservative as possible.

For the extinction projections, we started with 1500 fish (approximate population estimate for the Open system) spread among states according to the stable stage distribution for the reference matrix. We then projected population numbers using N_i,t+1_ = A*_i_*·N_i,t_, where N was a vector of population size for each state at time *t* and A*_i_* was the matrix for one of the *i* scenarios. To generate distributions of years to extinction, we determined times to extinction for 1000 matrices (A*_i_*) for each scenario. Matrices were generated using the parametric bootstrap approach outlined in Supplemental Text S1. We report years to extinction as the empirical cumulative frequency distributions that described the proportion of observations generating extinction times of x years or fewer.

#### 

##### Open system extinction

For each of the six scenarios and the reference matrix, we report averages and 95% confidence intervals for λ based on the 1000 bootstrap samples and the percentage of the 1000 runs for each scenario that resulted in a λ<1. We also report empirical cumulative frequency distributions for Open system extinction times for the reference matrix and each of the six scenarios as above and the number of years at 90 and 95% of the cumulative distributions.

##### Tributary extinction times

To provide an indication of extinction confidence times, we report years to tributary extinction for 90 and 95% cumulative frequency distribution values (i.e. 90 or 95% of the observations are less than x years). Tributary extinction times are independent of whether fish are removed or redistributed, so we only report times for the three removal scenarios.

##### Rescue by immigration

We estimated the number of immigrants required to ‘rescue’ the populations from extinction under each of the six scenarios for the Open system. For each scenario, we added a constant number of individuals to the population as N_i,t+1_ = A*_i_*·N_i,t_+M·w, where M was a multiplier ranging from 0 to 840 (step size 0.12) and w was the stable stage distribution of the reference matrix. For each time step, λ was calculated as N_t+1_/N_t_ and the final λ for a particular value of M was retained when λ_t+1_−λ_t_<10^−6^. We report the value of M that first returned a λ of one for each scenario. We also report the proportion of the initial population size (1500) for the immigration level that produced a λ = 1.

##### Rescue by demography

To determine whether the Open system can be demographically rescued from extinction by altering the stage 0 survival estimates, we replaced the Open system stage 0 survival with the Isolated stage 0 value. Then, as above, we estimated means and confidence intervals for λ and the percentage of runs with λ<1 for the six scenarios over 1000 parametric bootstrap runs.

## Supporting Information

Text S1 (0.03 MB DOC)

Figure S1 (0.03 MB TIF)

Figure S2 (0.01 MB TIF)

Table S1 (0.07 MB DOC)

Table S2 (0.03 MB DOC)

Table S3 (0.03 MB DOC)
