# Supplementary material for: Population Response to Habitat Fragmentation in a Stream-Dwelling Brook Trout Population
Source: PLoS One. 2007 Nov 7;2(11):e1139. doi: 10.1371/journal.pone.0001139 (PMC2190617; doi:10.1371/journal.pone.0001139)
Supplement: Text S1 — (0.03 MB DOC) [file pone.0001139.s001.doc]

Supplemental text S1

**Parametric bootstrap***.* To provide distributions of λ for each fragmentation scenario and for the unaltered matrices, we resampled matrices describing each scenario 1,000 times and calculated λ and confidence intervals for each realization. We incorporated parameter estimate variances in one of three ways depending on the data source for each matrix entry: 1) unknown, estimated variances with unknown covariance structure, 2) known variances with unknown covariance structure, and 3) known variance with known covariance structure. 1) Standard deviations and covariances for stage 0 survival were unknown; standard deviations were assumed to be 10% of the mean. For each bootstrap sample, deviates were drawn from a normal distribution, with uncorrelated means and standard deviations based on the 10% CV. 2) For fecundity estimates, we have good estimates of the means and standard deviations, but no information on the covariance structure. Fecundity means and standard deviations were calculated for median sizes of each size state based on the fish size-fecundity relationship using the nlpredci function in Matlab. 3) For the remaining matrix transition entries (estimated using the multi-state modeling), we had data on both variances and covariance structure. To provide deviates for the transition entries derived from the Ψ parameters that avoided unrealistic combinations of parameter values, we incorporated the variance-covariance matrix of the logit link function parameter estimates provided by M-Surge into a multivariate normal distribution. We used the logit link function values (‘beta’ or ‘mathematical’ values) rather than the real estimates because the beta values are distributed approximately normal while the real estimates are not (bounded 0 to 1). The parameter optimization procedure in M-Surge uses beta values during optimization. The beta values are then backtransformed to real parameter estimates. Mean beta values for the multivariate normal distribution were those that generated the unaltered matrix. Deviates from the multivariate normal distribution were generated using the Matlab script norm_rnd (available at www.spatial-econometrics.com). Deviates based on the beta values were back-transformed to real estimates (real=exp(beta)/(1+exp(beta)) prior to placing in the matrix entries.
