# Supplementary material for: Population Response to Habitat Fragmentation in a Stream-Dwelling Brook Trout Population
Source: PLoS One. 2007 Nov 7;2(11):e1139. doi: 10.1371/journal.pone.0001139 (PMC2190617; doi:10.1371/journal.pone.0001139)
Supplement: Table S1 — (0.07 MB DOC) [file pone.0001139.s004.doc]

Table S 1. Elasticities (% of total) for each entry in the reference matrix (Table 1).

|  |  | WB0 | OS0 | OL0 | WB1 | WB2 | WB3 | WB4 | OS1 | OS2 | OS3 | OS4 | OL1 | OL2 | OL3 | OL4 |
| --- | --- | --- | --- | --- | --- | --- | --- | --- | --- | --- | --- | --- | --- | --- | --- | --- |
|  |  | 1 | 2 | 3 | 4 | 5 | 6 | 7 | 8 | 9 | 10 | 11 | 12 | 13 | 14 | 15 |
| F WB | 1 | 0 | 0 | 0 | 0.16 | 0.21 | 0.40 | 3.51 | 0 | 0 | 0 | 0 | 0 | 0 | 0 | 0 |
| F OS | 2 | 0 | 0 | 0 | 0 | 0 | 0 | 0 | 0.06 | 0.08 | 0.17 | 1.19 | 0 | 0 | 0 | 0 |
| F OL | 3 | 0 | 0 | 0 | 0 | 0 | 0 | 0 | 0 | 0 | 0 | 0 | 0.09 | 0.15 | 0.31 | 1.30 |
| WB1 | 4 | 4.28 | 0 | 0 | 2.87 | 0 | 0 | 0 | 0 | 0 | 0 | 0 | 0.07 | 0 | 0 | 0 |
| WB2 | 5 | 0 | 0 | 0 | 2.87 | 2.08 | 0 | 0 | 0 | 0.01 | 0 | 0 | 0.06 | 0.03 | 0 | 0 |
| WB3 | 6 | 0 | 0 | 0 | 1.16 | 2.13 | 3.26 | 0 | 0 | 0.06 | 0.05 | 0 | 0 | 0.06 | 0.08 | 0 |
| WB4 | 7 | 0 | 0 | 0 | 0.04 | 0.44 | 2.94 | 27.82 | 0 | 0 | 0.13 | 1.47 | 0 | 0 | 0 | 0.32 |
| OS1 | 8 | 0 | 1.49 | 0 | 0 | 0 | 0 | 0 | 0.97 | 0 | 0 | 0 | 0 | 0 | 0 | 0 |
| OS2 | 9 | 0 | 0 | 0 | 0.01 | 0.04 | 0 | 0 | 1.04 | 0.75 | 0 | 0 | 0 | 0 | 0 | 0 |
| OS3 | 10 | 0 | 0 | 0 | 0.02 | 0.02 | 0.08 | 0 | 0.37 | 0.83 | 1.10 | 0 | 0 | 0 | 0 | 0 |
| OS4 | 11 | 0 | 0 | 0 | 0 | 0.05 | 0.08 | 1.09 | 0.03 | 0.09 | 0.96 | 5.58 | 0 | 0 | 0 | 0.53 |
| OL1 | 12 | 0 | 0 | 1.85 | 0.04 | 0 | 0 | 0 | 0 | 0 | 0 | 0 | 1.78 | 0 | 0 | 0 |
| OL2 | 13 | 0 | 0 | 0 | 0.02 | 0.05 | 0 | 0 | 0 | 0 | 0 | 0 | 1.49 | 1.84 | 0 | 0 |
| OL3 | 14 | 0 | 0 | 0 | 0.03 | 0.03 | 0.05 | 0 | 0 | 0 | 0 | 0 | 0.18 | 1.32 | 3.23 | 0 |
| OL4 | 15 | 0 | 0 | 0 | 0 | 0 | 0 | 0.75 | 0 | 0 | 0 | 0.18 | 0 | 0 | 1.23 | 10.93 |
