# Supplementary material for: Population Response to Habitat Fragmentation in a Stream-Dwelling Brook Trout Population
Source: PLoS One. 2007 Nov 7;2(11):e1139. doi: 10.1371/journal.pone.0001139 (PMC2190617; doi:10.1371/journal.pone.0001139)
Supplement: Table S2 — (0.03 MB DOC) [file pone.0001139.s005.doc]

Table S 2. Elasticities (% of total) for each entry in the reference matrix for Isolated tributary (Error: Reference source not found).

|  |  | 1 | 2 | 3 | 4 |
| --- | --- | --- | --- | --- | --- |
| F | 0 | 0.91 | 1.34 | 1.38 | 5.92 |
| 1 | 9.55 | 13.33 | 0 | 0 | 0 |
| 2 | 0 | 8.39 | 10.60 | 0 | 0 |
| 3 | 0 | 0.25 | 6.83 | 7.53 | 0 |
| 4 | 0 | 0 | 0.23 | 5.70 | 28.06 |
