# Supplementary material for: Population Response to Habitat Fragmentation in a Stream-Dwelling Brook Trout Population
Source: PLoS One. 2007 Nov 7;2(11):e1139. doi: 10.1371/journal.pone.0001139 (PMC2190617; doi:10.1371/journal.pone.0001139)
Supplement: Table S3 — (0.03 MB DOC) [file pone.0001139.s006.doc]

Table S 3. Average and confidence intervals of λ and percentage of 1000 runs with λ < 1 for the Open system seven scenarios incorporating the stage 0 early survival from the Isolated tributary (0.04875 from Error: Reference source not found replaced 0.03356 in Error: Reference source not found).

|  |  |  |  |  |  |  |
| --- | --- | --- | --- | --- | --- | --- |
|  |  | Average λ [95% C.I.] | |  | Percent of runs with λ < 1 |  |
| 1 | Reference | 1.0322 | [1.0196; 1.0440] |  | 0 |  |
| 2 | Remove OL | 1.0155 | [0.9987; 1.0310] |  | 0.4 |  |
| 3 | Remove OS | 1.0095 | [0.9947; 1.0243] |  | 6.8 |  |
| 4 | Remove Both | 0.9930 | [0.9768; 1.0103] |  | 90.4 |  |
| 5 | Redistribute OL | 1.0320 | [1.0163; 1.0480] |  | 0 |  |
| 6 | Redistribute OS | 1.0255 | [1.01138; 1.0390] |  | 0 |  |
| 7 | Redistribute Both | 1.0236 | [1.0057; 1.0438] |  | 0 |  |
